# Correction: Do birthrates contribute to sickness absence differences in women? A cohort study in Catalonia, Spain, 2012–2014

**DOI:** 10.1371/journal.pone.0245332

**Published:** 2021-01-06

**Authors:** Andrew N. March, Rocío Villar, Monica Ubalde-Lopez, Fernando G. Benavides, Laura Serra

In the Funding statement, a grant number from the funder the Health Institute Carlos III–Subdirection General of Evaluation and Promotion of Investigation is missing. The grant number is: FIS WORKSS PI17/00220.
