# Supplementary material for: Do birthrates contribute to sickness absence differences in women? A cohort study in Catalonia, Spain, 2012-2014
Source: PLoS One. 2020 Aug 26;15(8):e0237794. doi: 10.1371/journal.pone.0237794 (PMC7449461; doi:10.1371/journal.pone.0237794)
Supplement: Attachment — Submitted filename: PLOSONE Reviewer Responses_final.docx [file pone.0237794.s002.docx]

Dear Editor,

Thank you for the opportunity to submit for your review a revised and edited manuscript. We appreciate the reviewer’s comments as they have contributed to making this a stronger and more comprehensive research article. Below, we provide responses to the reviewer comments and detail how they were addresses in the manuscript. Responses to the reviewers are indented and italicized.

**5. Review Comments to the Author**

Please use the space provided to explain your answers to the questions above. You may also include additional comments for the author, including concerns about dual publication, research ethics, or publication ethics. (Please upload your review as an attachment if it exceeds 20,000 characters)

**Reviewer #1**: The aim of the study was to investigate differences in sickness absence rates between different age groups with different fertility rates.
However there are some important methodological problems and the study do not add much knowledge to the area.

*We thank the reviewer for this comment. The methodological issues mentioned have been responded to below in comments about specific methodological issues. We would argue that the research presented in this article does add to the knowledge on SA since very little research has been performed on the topic in Spain or in other Southern European countries that exhibit different employment characteristics than other countries where more research has been done. Additionally, this study investigates SA in a unique social context to Spain, in which domestic or reproductive work is still largely performed by women despite their increasing involvement in the labor market. Moreover, a compelling contribution of this study is in identifying the diagnoses for which the increased risk of SA is substantial. Identifying the diagnoses that contribute most to the overall effect of increased risk of SA in younger women can help inform allocation of healthcare resources, mitigation strategies in the workplace, and policy aimed to guard workers’ health. This argument for the importance of this study has been made more explicit in the article.*

*Added text: (Lines 100-102) “Due to the differences in sickness absence benefits between countries, it is not clear whether research findings from other countries are applicable to the Spanish worker.”*

*(Lines 118-119) “Overall, Spain is characterized as having lower fertility and an older age at first birth than the rest of the EU”*

*(Lines 125-129) “Moreover, a broad description of SA across age and disease states is, at the time of this publication, insufficient to understand the potential causes of this gender gap in a Southern European state such as Spain, which has a weaker welfare state than Northern European countries, where this phenomenon is better described.”*

Their dependent variable is numbers of sickness absence spells given as IR (numbers of SA spells/100 employment years).
The independent variable is three age groups with different fertility rates.

I have two main concerns regarding the methodology:

1)
How do they count employment years. No information is given regarding censoring and do they take into account other reasons for absence ie maternity leave. What about the duration of SA spells? Could it be, that older women have longer spells and thus fewer numbers of spells?
Risk time in the different is not stated anywhere - not in the tables nor in the text.

*We appreciate the reviewer’s comments. One strength of this study is that we have been able to estimate the real person-years at risk, allowing for a longitudinal view of SA behavior and a more precise calculation of incidence. The methods for calculating person-years at risk are described in the methods section (lines 190-192). Censoring due to an interrupted affiliation to Social Security or due to SA is mentioned in the methods section. As the reviewer asks, the actual person-years at risk have now been added to Table 1.*

*Because the dataset did not include information on pregnancy or maternal leave, it was not possible to subtract time using maternal leave from calculations of person-years at risk. However, the resultant underestimation of incidence rates will be more prominent in the younger age ranges since they have higher birthrates, and thus reinforces the results obtained. The duration has not been considered in this analysis, it will be the subject of a future research.*

*In order to discuss whether length of SA may explain differences in the incidence of SA, median duration of SA was added to Table 2 on line 230. Subsequently, in the discussion, it is mentioned that SA episodes for older women are modestly longer than for younger age groups, but since there is not a similar yet inverse trend of increasing duration with the continuous decline in incidence of SA with older age, whether duration could be a factor requires further analysis.*

*Added text: (Lines 226-229) “Though the median duration of SA varies widely depending primarily on the diagnostic group, overall SA episodes are longer for women in the late reproductive group than for early- or middle-reproductive groups”*

*(Lines 377-383) “Additionally, subsequent research should investigate if the longer duration of SA episodes in late-reproductive women observed in this study is a contributing factor to the decrease in SA incidence with decreasing birthrates. However, this current study does not initially seem to support that relationship, as the trend of decreasing incidence of SA with lower birthrates is not mirrored by an increasing trend in SA duration. Rather an increase in SA duration is only observed in the late-reproductive group.”*

2)
They have no knowledge of pregnancies among the women, thus the independent variable is only a proxy

*We agree with the reviewer's comment and, in fact, this is already acknowledged in the limitations. At the same time, the percentages of SA due to obstetric causes mirror the same trend in birthrates, leading us to conclude that it is a well-constructed proxy variable. Though we remain conservative in our conclusions and tie them back to birthrates. We also acknowledge that pregnancy, insofar that it is closely linked to birthrates, may be a contributing factor based on the disease-specific IRs observed in this study as well as previous literature that describes diseases common in pregnancy.*

The methods section is very short and not sufficient informative. There is no flow chart explaining how the final population was reached and it is sparsely described in the text. At what dates do the study start and end?

*We appreciate the reviewer’s comment, and have accordingly added a flowchart (Figure 1) on line 144 that depicts both the construction of the WORKss cohort from the two administrative datasets, as well as the exclusion of individuals, resulting in the final sample used in the study. Additionally, the methods section has been complemented with more information on the study period, the databases used, clearer definition of SA, and explanation for the statistical models used.*

*Added text: (Lines 137-138) “The study period went from the beginning of 2012 to the end of 2014.”*

*(Lines 148-156) “The annual reference population for the MCVL is any individual who has contributed to or received payments from Social Security at any point during that year. Individuals are selected to the MCVL based on a simple random sample, and remain a part of the MCVL indefinitely until they contribute to or receive from the Social Security at any point over a full calendar year. New individuals enter the MCVL every year until the sample reaches 4% of the population. While the MCVL is a robust sample that allows for the inclusion of individuals with infrequent employment, it excludes some categories of civil servants, which represent about 5% of the Spanish working population.”*

*(Lines 163-164) “An SA spell was defined as any absence from work due to common disease as certified by a physician, as is required by law.”*

*(Lines 198-202) “Incidence risk ratios (IRR) were calculated using crude and adjusted Poisson and negative binomial models, with the data presented deriving from whichever of the two demonstrated the higher log likelihood. Consequently, IRR values are based on Poisson models for overall SA, and negative binomial models were used for specific diseases in order to compare the incidence rates of SA among age groups.”*

Regarding results:
There is no table 1 describing the different age groups with regard to contract types, occupations, country of origin, mean age, mean duration of SA spells, risk time, numbers of women etc.

*We appreciate this reviewer comment. According to this suggestion, a table with descriptive statistics was added to the manuscript on line 216 as Table 1. This table provides median age of each group, and the distribution of occupational and demographic characteristics according to the different age groups.*

Regarding conclusion
They conclude that results suggest that differences in sickness absence between early- and late reproductive groups can largely be explained by higher incidence of infectious, obstetric and musculoskeletal disorders in younger women with (they write lower birthrates, I think they mean higher birth rates) higher birthrates.

*As the reviewer suggests, the sentence was intended to read “higher birth rates” and has accordingly been edited.*

*Edited text: (Line 410) “In summary, these results suggest that the differences in SA between early- and late-reproductive-aged women can largely be explained by the higher incidences of infectious, obstetric, and musculoskeletal diseases in younger working women with higher birthrates.”*

First the overall IRR in the fully adjusted model is 1.04, which in this study with many uncertainties, is not compelling.

*Following the reviewer’s suggestions, edits have been made to clear up the methodological uncertainties that were present in the article, and give greater confidence in the results presented. While an IRR of 1.04 may not be a dramatically increased risk, it is a significantly larger risk than that of the reference group. Moreover, the most compelling contribution of this study is in identifying the diagnoses for which the increased risk of SA is substantial. Identifying the diagnoses that contribute most to the overall effect of increased risk of SA in younger women can help inform allocation of healthcare resources, mitigation strategies in the workplace, and policy aimed to guard workers’ health.*

*(Lines 163-164) “An SA spell was defined as any absence from work due to common disease as certified by a physician, as is required by law.”*

*(Lines 173-177) “After excluding women under 25, because they had relatively low birth and employment rates (8.76 births/1000 persons and below 25%, respectively), and women over 54 (natality data not reported), 16,482 women were removed and the final sample size was 47,879 women, or about 4.2% of the female working population between 25 and 54 years of age in Catalonia.”*

*(Lines 198-202) “Incidence risk ratios (IRR) were calculated using crude and adjusted Poisson and negative binomial models, with the data presented deriving from whichever of the two demonstrated the higher log likelihood. Consequently, IRR values are based on Poisson models for overall SA, and negative binomial models were used for specific diseases in order to compare the incidence rates of SA among age groups.”*

Second the found difference in IR of diseases related to pregnancy is not surprising, but I am not convinced that it can explain the difference, if any, in overall SA.

*A sentence has been added in the discussion to more completely explain why this may be the case. In this sentence, ]we argue that given the high incidence of these diagnoses in younger women and relatively low incidence in older women, in addition to the higher IR in younger women, is a compelling reason to suggest that pregnancy may, in part, contribute to the higher overall IRR in younger women.*

*Added text: (Lines 350-354) “Additionally, the high incidence of SA in early-reproductive age due to obstetric diagnoses and low back pain coupled with the increased risk observed in early-reproductive age women, potentially due to pregnancy, may partially explain the higher overall IRR in early-reproductive women compared to late-reproductive women.”*

**Reviewer #2: General comments**:
This study is focusing on a topic that is becoming increasingly important as female employment rates are growing in many countries. The study does provide some interesting findings.

However, I have some major concerns:

• A major limitation is that the analyses do not include information about working hours, or part time vs full time employment. Since the extent of part time employment is widespread among women, this might be an important confounder, which has received no attention at all.

*We thank the reviewer for this comment and share their interest in analyzing how the addition of this covariable could have allowed for a more detailed interpretation of the results. Unfortunately, this variable was not available to us at the time this research was conducted. However, we were able to include information on the type of contract and occupational category that similarly allow for a detailed description of occupational conditions. We will take this comment into consideration when designing future research.*

• It is not clear how the study brings new knowledge to the research field.

*As mentioned in response to Reviewer 1, a description of how this is some of the first longitudinal research on SA being performed on a population in Southern Europe is discussed as a principle strength of this article in the discussion section. Text has been added in the introduction on how this research provides novel insights into SA presented in the context of a unique welfare state and socioeconomic environment in Spain.*

*Added text: (Lines 100-102) “Due to the differences in sickness absence benefits between countries, it is not clear whether research findings from other countries are applicable to the Spanish worker.”*

*(Lines 118-119) “Overall, Spain is characterized as having lower fertility and an older age at first birth than the rest of the EU”*

*(Lines 125-129) “Moreover, a broad description of SA across age and disease states is, at the time of this publication, insufficient to understand the potential causes of this gender gap in a Southern European state such as Spain, which has a weaker welfare state than Northern European countries, where this phenomenon is better described.”*

• The study is generally marked by a lack of precision. For example, it is unclear whether all sickness absence spells are included, or whether spells are recorded after a certain number of days.

*This specific concern of SA spell inclusion has been expanded upon in the introduction section, as well as reorganized to more clearly describe the process of recognizing a SA episode in chronological terms.* *Additionally, an explicit definition of SA for the purposes of this study was provided in the methods section.*

*Added text: (Lines 85-93) “The sickness absence benefit in Spain is a social benefit from which any worker can benefit given that they have been affiliated to the Social Security System. For common disease, not occupational injury or disease, current legislation requires that the sick leave be certified by a National Health System physician in order to offer a diagnosis and recognize the absence from work as SA. SA spells are recorded for all health-related absences recognized by a National Health System physician beginning on the day the physician certifies the absence until the health condition initiating the absence is resolved, or in extreme instances, when the condition cannot be resolved and permanent disability is required.”*

*(Lines 163-164) “An SA spell was defined as any absence from work due to common disease as certified by a physician, as is required by law.*

• The study lacks important information about the quality of the research data.

*More information was provided on the databases used in this study under the study population section in methods to offer more details on the quality of the data. In this addition, the rigors of the methodological process behind the databases are described. A citation to a published description of the cohort used in this study was also provided to allow the reader to review the quality of the data in depth. Additionally, as Reviewer 1 suggested, a flowchart (Figure 1) depicted the construction of the cohort used in the study and the final sample analyzed was included on line 144.*

*Added text: (Lines 142-143) “A detailed description of the WORKss Cohort has previously been described by López Goméz and colleagues.”*

*(Lines 148-156) “The annual reference population for the MCVL is any individual who has contributed to or received payments from Social Security at any point during that year. Individuals are selected to the MCVL based on a simple random sample, and remain a part of the MCVL indefinitely until they contribute to or receive from the Social Security at any point over a full calendar year. New individuals enter the MCVL every year until the sample reaches 4% of the population. While the MCVL is a robust sample that allows for the inclusion of individuals with infrequent employment, it excludes some categories of civil servants, which represent about 5% of the Spanish working population.”*

*Edited text: (Lines 175-177) “After excluding women under 25, because they had relatively low birth and employment rates (8.76 births/1000 persons and below 25%, respectively), and women over 54 (natality data not reported), 16,482 women were removed and the final sample size was 47,879 women, or about 4.2% of the female working population between 25 and 54 years of age in Catalonia”*

Specific comments:

Abstract:

26
Please clarify this aim. What is meant by "distribution of sickness absence trends"? Sickness absence differentials between age groups? Trends in sickness absence across age groups? It is not clear what the term “distribution” contributes with in this aim.

*With the intention to answer the reviewer's doubt, “distribution of” was changed to “differences in” for clarity*

30
What is the percentage? (How many percent do the 47879 female employees constitute of the total population of female employees in the current age groups during this period of time?)

*Data was added indicating that about 4.2% of the reference population is included in the final sample. However, this information was provided in the methods section as it seemed too detailed to provide in the abstract. Additionally, a flowchart (Figure 1) describing how the final sample was determined was included on line 144.*

*Added text: (Lines 173-177) “After excluding women under 25, because they had relatively low birth and employment rates (8.76 births/1000 persons and below 25%, respectively), and women over 54 (natality data not reported), 16,482 women were removed and the final sample size was 47,879 women, or about 4.2% of the female working population between 25 and 54 years of age in Catalonia.”*

32
It seems a bit odd that the study excludes women younger than 25, when women as old as 54 are included. Please clarify. Also, the age groups are quite large, is it possible to make them smaller? For example 25-29 in one group, and 30-35 in the next?

*As described later in the text, women younger than 25 have low birth rates and low employment. Women ages 20-24 had a birth rate of 8.76 births/1000 persons (less than half that for women 35-44). The employment rate in women 16-24 in the population was around 25% at the time of the study and the unemployment rate was around 50% (more than double that of women 25-54). Especially regarding the high unemployment and low employment, we considered adding this age group of women would add a severe selection bias to the study since such a small portion of the population able and looking for work was actually able to find work. Furthermore, information about birth rates from the Catalan Statistic Institute are organized by these age groups.*

34
Do these registries not include information about working hours, or the proportion of part time /full time employment among these women? This is particularly important when analyzing the impact of fertility on sickness absence among women, because longer working hours probably apply more often to younger women who do not have children.

*We again appreciate this reviewer’s comment, but as it is the same as the first comment made by this reviewer, it is addressed above.*

Introduction:

56
What kind of legislation? EU-legislation? Legislation in Catalonia? It is not clear if legislation actually has been changed, or if there simply is a growing recognition of the need for doing so. How is this study related to the (need for) changing legislation?

*It was clarified to say Spanish legislation, similar to that in other European countries. The implications this research has on policy was added to the end of the discussion.*

*Added text: (Lines 416-418) “Building in more flexibility to current maternity leave policies would give pregnant women more options in managing their health during pregnancy, and could reduce dependence on SA.”*

70
Is this the kind of legislation that were mentioned in the first paragraph? In that case, the legislation should not be introduced until it is addressed more fully, that is, in the third paragraph.

*According to the reviewer’s comment, the mention of legislation in the first paragraph was removed so that the discussion on legislation can be focused on in the third paragraph.*

77
Is confusing that the term "SA" refers to both "sickness absence" (being away from work) and "sickness absence benefit" (social insurance benefit). Please use different terms to distinguish between these.

*We appreciate the reviewer’s comment and to clarify the understanding of the text we have modified it accordingly. Then, because the benefit is referred to only briefly in the text, and not frequently, “SA” is used to refer to “sickness absence” and the full term “sickness absence benefit” is used when referring to the social benefit.*

84-85
What does it mean that spells are "reported"? Is that the same as being recorded in the registry that your study applies? What separates spells that are recognized by a National Health Service physician from spells that are not? Is the physician involved even the first few days when the worker gets no compensation, after 3 days when the employer covers the employment, or from day 17 when the National Social Security covers the pay, or at some other time? If spells are not recorded in the registry from the first sick day, but rather after a particular length of duration, please consider if this implies that the dependent variable in this study is truncated, and if so, if other regression models are more suitable than Poisson and negative binomial regression.

*The word “reported” was exchanged for “recorded,” which is a more accurate representation of the data collection. The paragraph explaining the recognition of SA was reorganized for clarity, beginning with the recognition of the absence, and then turning to the compensation. This way the flow of the sentence is more chronological to the reader. Additionally, when a SA episode begins and ends was clarified in the text. SA begins on the day the absence is recognized by a physician, until the health condition is resolved.*

*Added text: (Lines 85-96) “The sickness absence benefit in Spain is a social benefit from which any worker can benefit given that they have been affiliated to the Social Security System. For common disease, not occupational injury or disease, current legislation requires that the sick leave be certified by a National Health System physician in order to offer a diagnosis and recognize the absence from work as SA. SA spells are recorded for all health-related absences recognized by a National Health System physician beginning on the day the physician certifies the absence until the health condition initiating the absence is resolved, or in extreme instances, when the condition cannot be resolved and permanent disability is required. After the third day of absence, the employee’s wages are covered between 60% and 70%, first by the employer, until the sixteenth day of absence when the National Social Security Institute begins to pay until the end of the SA episode.”*

99
In the Abstract there seem to be one aim specified, but in line 99 there seem to be several aims. Please be consistent.

*The aim is the same in the abstract and at the end of the introduction, but use different wording. The text was edited to clarify that the singular aim is the same in both places.*

*Edited text: (Lines 26-27) “This study explores the differences in sickness absence trends in women according to reproductive age group and medical diagnoses.”*

*(Lines 129-132) “The aim of this study is to compare the incidence rates of overall SA and according to specific diseases among early- middle- and late-reproductive age groups in female workers in Catalonia (Spain) between 2012 and 2014.”*

Please provide more information about any maternity leave scheme, the overall fertility rate, mean age for first-time pregnant women, and the level of employment among pregnant women in Catalonia.

*On the advice of the reviewer, a paragraph including the requested information was added lines 111-119 as well as a brief description of how these data relate to the EU as a whole, to emphasize that Spain has unique employment characteristics and circumstances surrounding pregnancy that demand research on SA in the Spanish context.*

*Added text: (Lines 111-119) “In Spain, maternity leave benefits provide women 16 continuous weeks of leave paid at 100% of their normal salary. Six of these weeks must be used directly after birth, but the remaining 10 weeks may be used before or after the birth, donated to the woman’s partner, or any combination thereof. In 2014, of those who provided a response, 53% of women who gave birth in Catalonia described themselves as being active in the labor market. In that same year, the total fertility in Spain was 1.32, and a 2015 estimate suggests that the median age of the mother at first birth was 30.7 years for Spain. Overall, Spain is characterized as having lower fertility and an older age at first birth than the rest of the EU.”*

Methods:

104
Please give more details about the quality of the data. What is the purpose of these registries? What is the level of reliability? Are the registries characterized by any weaknesses?

*We thank the reviewer for providing this helpful suggestion. Reviewer 1 provided a similar comment that was also addressed above. Additional data on the MCVL was added to explain the methodological processes that are used to construct the sample, and how that lends to its robustness. The predominant limitation of not including some civil servants was mentioned.*

*We have added this text to the manuscript in order to clarify this question:*

*Added text: (Lines 148-156) “The annual reference population for the MCVL is any individual who has contributed to or received payments from Social Security at any point during that year. Individuals are selected to the MCVL based on a simple random sample, and remain a part of the MCVL indefinitely until they contribute to or receive from the Social Security at any point over a full calendar year. New individuals enter the MCVL every year until the sample reaches 4% of the population. While the MCVL is a robust sample that allows for the inclusion of individuals with infrequent employment, it excludes some categories of civil servants, which represent about 5% of the Spanish working population.”*

119
Does the dependent variable not account for the wide extent of part time employment among Spanish women? Full time employed women will probably have many more SA spells than part time employed women. This should be accounted for in the analyses. If it is not possible to account for this, the implications for the findings should be thoroughly discussed.

*As the reviewer has mentioned previously, this is an important limitation. In the response to this reviewer’s first comment, we address why we were unable to include this variable. According to the reviewer’s comment, text was added to the discussion to explain this limitation.*

*Added text: (Lines 393-396) “Furthermore, data on full-time or part-time employment was not available for this study. Those who are employed full-time may be expected to incur more SA, though type of contract provides similar information about the occupational context, and was able to be analyzed in the study.”*

Also, it is unclear if it includes all possible sickness absence spells, or only spells recognized by the National Health Service physicians.

*An explicit definition of SA for the purposes of this study was added.*

*Added text: (Lines 163-164) “An SA spell was defined as any absence from work due to common disease as certified by a physician, as is required by law.”*

144
This seems a bit odd because "affiliate-years" is not a widely accepted term. Please use more common language.

*According to the reviewer’s comment, “affiliate-years” has been changed to “person-years” throughout the article*

152
Please explain why these types of regression (Poisson and the negative bionomial) were applied.

*An explanation on comparing log likelihoods was added to clarify the criterion that was used in determining the use of Poisson or negative binomial models.*

*Added text: (Lines 198-200) “Incidence risk ratios (IRR) were calculated using crude and adjusted Poisson and negative binomial models, with the data presented deriving from whichever of the two demonstrated the higher log likelihood. Consequently, IRR values are based on Poisson models for overall SA, and negative binomial models were used for specific diseases in order to compare the incidence rates of SA among age groups.”*

158
Please provide some more information about the ethics approval. Is this paper part of a larger study that was approved by the Committee? If so, what kind of study is that?

*Text has been edited to specify that the ethics approval was specific to the work carried out for this article.*

*Edited text: (Line 206) “The protocol for the study described in this article (2018/8036/I), was approved by the Clinical Ethics Committee at Parc de Salut Mar in Barcelona, Spain.*

Results:

163-165
Please consider the possibility that age differentials in SA partly result from age specific patterns in part-time employment. Considering that female employment has increased in Spain recently, one might expect that younger women are more likely to longer working hours than older women, (particularly if the latter group more often have children). If so, one would expect younger women to have higher rates of sickness absence, simply because they work longer hours. This implies that working hours is an important possible confounder that should be included in the analyses. If that is not possible, the possible implications of this limitation should be properly discussed.

*We once again thank the reviewer for bringing this limitation to light. Our response to the content of this comment can be found under this reviewer’s first comment. Likewise, as noted in a previous response to a similar comment made by this reviewer, a discussion of part-time employment has been added to the limitations.*

*Added text: (Lines 393-396) “Furthermore, data on full-time or part-time employment was not available for this study. Those who are employed full-time may be expected to incur more SA, though type of contract provides similar information about the occupational context, and was able to be analyzed in the study.”*

It is confusing that the results are characterized as “significant” in the text, as statistical significance is not easily detectable from the tables. For example, it is possible that overlapping confidence intervals yield significantly different point estimates (IRs). I would recommend an extra column with a p-value for the trend (early-middle-late).

*We appreciate the reviewer's comment, however, in regards to demonstrating a significant trend, we believe that using confidence intervals helps to demonstrate the significance of the trends in a more illustrative manner. Providing the confidence intervals can clearly demonstrate that the intervals in which we expect the IR estimates to fall, do not overlap between the different age groups. For example, for low back pain, the inclusion of confidence intervals clearly demonstrates that the IRs for the different age groups are not expected to be the same in any of the groups and that there is a trend of decreasing SA due to low back pain with older age.*

Discussion:

327
If the study’s main contribution to the research field is that it provides empirical analyses from Southern Europe, this should be included and elaborated on in the introduction.

*We thank the reviewer for this observation. Accordingly, and also in response to a similar comment made my Reviewer 1, a sentence describing the need for research on this subject in a Southern European country was inserted lines 118-119. Additionally, a sentence detailing the inability to rely on research performed in other countries in order to understand sickness absence in Spain was added lines 100-102.*

*Added text: (Lines 100-102) “Due to the differences in sickness absence benefits between countries, it is not clear whether research findings from other countries are applicable to the Spanish worker.”*

*(Lines 118-119) “Overall, Spain is characterized as having lower fertility and an older age at first birth than the rest of the EU”*

335
According to the above text, younger working women have higher, not lower birthrates (which is stated here).

*Text was corrected to state that younger women exhibit higher birthrates. Reviewer 1 made the same comment and it was addressed accordingly.*

*Edited text:* *(Line 410) “In summary, these results suggest that the differences in SA between early- and late-reproductive-aged women can largely be explained by the higher incidences of infectious, obstetric, and musculoskeletal diseases in younger working women with higher birthrates.”*
