# Supplementary material for: Do birthrates contribute to sickness absence differences in women? A cohort study in Catalonia, Spain, 2012-2014
Source: PLoS One. 2020 Aug 26;15(8):e0237794. doi: 10.1371/journal.pone.0237794 (PMC7449461; doi:10.1371/journal.pone.0237794)
Supplement: Attachment — Submitted filename: Reviewer Response 7.12.20.docx [file pone.0237794.s003.docx]

*We thank the reviewer for these additional comments, which are greatly helpful to making the manuscript stronger and giving it more clarity. The responses to the reviewers comments are indented and italicized.*

Thank you for the very fine response.
I do think the manuscript has improved significantly with the changes and the added table.

Still, I would say that the overall risk of SA is (only) MODESTLY increased in the young age group. (1.01, 95CI 1.01-1.06). The small increase could very well be due to share of parttime workers or duration of SA spells or other confounding factors. Thus I would prefer the wording: A slightly
increased/a modestly increased risk (l.308-309, l.366 and other places).

However, the findings show the differences between age groups regarding reasons for SA, and it indicates that adjustment of work or allocation of healthcare ressources to pregnant workers can be necessary if wanting to reduce SA in the young age group.

*We appreciate this comment for bringing greater clarity to the findings. Correspondingly, we have added “modestly” to provide extra context to the results.*

*Edited text: (Lines 252-254) In Table 4, the crude model for overall SA shows that the IRR for the early-reproductive age group (IRR=1.09; 95% CI: 1.06-1.12) is modestly, yet significantly greater than the late-reproductive age group (IRR=1 [reference])*

*(Lines 311-313) In addition, the moderately increased risk of SA in early-reproductive aged women appears to be almost entirely explained by diagnostic groups and not occupational characteristics.*

Extra comments:

I would recommend to update the references regarding occupational exposure and health effects (ref 8).

*We thank the reviewer for this suggestion to update this reference. A more recent article was used to support the statement made, and the text was modified accordingly.*

*Edited text: (Lines 68-71) Evidence demonstrates the relation between occupational exposures and health effects during pregnancy, such as an increase in the number of low birth weight and prematurity in relation to a heavy physical workload (8).*

l.155 "excludes som categories of civil servants" (5%) - which categories? please give some examples if possible.

*This is an important clarification for those unfamiliar with the insurance systems in Spain. We have therefore defined the populations that are excluded and provided a few examples.*

*Edited text: (Lines 146-149) While the MCVL is a robust sample that allows for the inclusion of individuals with infrequent employment, it excludes some categories of civil servants covered by distinct insurance funds, such as university professors and military personnel (22), which represent about 5% of the Spanish working population*

The median duration of SA is in days, months? (please state it in the table and the text).

*Thank you for pointing out this oversight in labeling the variable units. The table legend was updated to include that the median durations are represented in days.*

*Edited text: (Line 227) MD: median duration of SA in days*
